# Cardiopulmonary arrest caused by airway obstruction due to acute transient thyroid swelling after fine-needle aspiration biopsy

**DOI:** 10.1186/s40981-023-00636-6

**Published:** 2023-07-13

**Authors:** Shuto Yoshizawa, Chiaki Nemoto, Satoki Inoue

**Affiliations:** 1 The Junior Resident Center, Ohara General Hospital, 6-1 Ohomachi, Fukushima, 960-8611 Japan; 2 Department of Anesthesiology, Ohara General Hospital, 6-1 Ohomachi, Fukushima, 960-8611 Japan; 3 Department of Anesthesiology, Fukushima Medical University, 1 Hikarigaoka, Fukushima, Fukushima 960-1295 Japan

**Keywords:** Fine-needle aspiration biopsy, Acute transient thyroid swelling, Airway obstruction

## To the Editor

Acute transient thyroid swelling (ATTS) is a complication of fine-needle aspiration biopsy (FNAB) and is clearly distinguished from hemorrhagic complications. We experienced a case of cardiopulmonary arrest caused by ATTS that resulted in severe airway obstruction.

An 81-year-old woman (height, 151 cm; weight, 45 kg) with no history of allergy or thyroid disease was found having a thyroid nodule with calcification and FNAB was scheduled. From the left lobe of the thyroid gland where the nodule with calcification was located, FNAB was performed with a 21-gauge needle without local anesthesia. The puncture was smoothly and uneventfully performed. About 90 min after FNAB, the patient felt discomfort while swallowing and then developed severe dyspnea. Three hours after FNAB, she developed cardiopulmonary arrest. Cardiopulmonary resuscitation was performed, and her trachea was intubated with inner diameter of a 6.0-mm intratracheal tube using videolaryngoscope. (McGrath MAC®; Aircraft Medical Ltd., Edinburgh, UK). At the time of intubation, we did not observe apparent edematous findings above and around the vocal cords. She was successfully resuscitated.

**Fig. 1:**
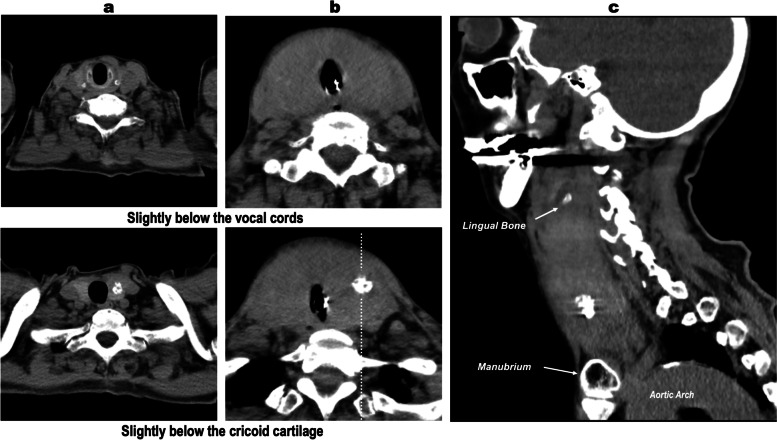
CT images of the thyroid gland **a** 3 weeks before fine-needle aspiration biopsy, **b** axial, and **c** sagittal views of the white dot line in **b**, immediately after tracheal intubation. **a** Three weeks before fine-needle aspiration biopsy. A mass with calcification is observed in the left lobe of the thyroid gland. The thyroid gland is hardly identified especially at slightly below the vocal cords. **b** Immediately after tracheal intubation. Diffuse swelling of the thyroid gland, especially at the subglottic level compared before biopsy. **c** Sagittal views of the white dot line in **b**. A swollen thyroid gland extending from the lingual bone developing beyond the manubrium of the sternum. No hemorrhage was observed

The ultrasound examination which was performed before cardiopulmonary arrest indicated that bilateral thyroid was diffusely swelling without hematoma. Her computed tomography findings are shown in Fig. 1. No hematoma was present, but bilateral massive swelling of the thyroid was observed. The next day (about 22 h after FNAB), although edema remained, the thyroid swelling had considerably improved compared with the day before and her trachea was extubated with no complications. Post-extubated course was smooth, and she was discharged from the hospital 4 days later. The result of FNAB did not indicate malignancy.

Most of the anesthesiologists recognize that acute upper airway obstruction caused by hematoma is a fatal complication after thyroid surgery, but ATTS is not widely known because, in most cases, it is self-limiting without airway obstructions [1]. In patients with ATTS, the thyroid diffusely swells, sometimes even increasing several folds in size. According to some reports, ATTS causes no airway obstruction and patients recover within several hours without special treatment [2, 3]. Even in the most severe cases, it has been suggested that ATTS is transient and self-limiting with no symptoms of airway obstruction [1]. However, some recent reports have indicated that swelling is not limited to the thyroid but also extends to the surrounding tissue and that the presence of airway obstruction requires tracheal intubation [4]. The mechanism of ATTS is not clearly understood, but release of a potent vasodilator substance with secondary vasodilation and capillary leakage has been suggested to constitute the underlying pathophysiological mechanism [5]. Therefore, the severity of ATTS may vary depending on the individual patient. We may need to reconsider the presumption that ATTS is safe and not life-threatening.

## Data Availability

Not applicable.

## Abbreviations

ATTS: Acute transient thyroid swelling
FNAB: Fine-needle aspiration biopsy
